# Intraneuronal β-Amyloid Accumulation: Aging HIV-1 Human and HIV-1 Transgenic Rat Brain

**DOI:** 10.3390/v14061268

**Published:** 2022-06-10

**Authors:** Hailong Li, Kristen A. McLaurin, Charles F. Mactutus, Benjamin Likins, Wenfei Huang, Sulie L. Chang, Rosemarie M. Booze

**Affiliations:** 1 Department of Psychology, University of South Carolina, Columbia, SC 29208, USA; hailong@mailbox.sc.edu (H.L.); mclaurik@email.sc.edu (K.A.M.); mactutus@mailbox.sc.edu (C.F.M.); likins@musc.edu (B.L.); 2 Institute of NeuroImmune Pharmacology, Seton Hall University, South Orange, NJ 07079, USA; wenfei.huang@shu.edu (W.H.); sulie.chang@shu.edu (S.L.C.); 3 Department of Biological Sciences, Seton Hall University, South Orange, NJ 07079, USA

**Keywords:** β-amyloid, prepulse inhibition, RNAscope, neurodegenerative diseases, HIV-1

## Abstract

The prevalence of HIV-1 associated neurocognitive disorders (HAND) is significantly greater in older, relative to younger, HIV-1 seropositive individuals; the neural pathogenesis of HAND in older HIV-1 seropositive individuals, however, remains elusive. To address this knowledge gap, abnormal protein aggregates (i.e., β-amyloid) were investigated in the brains of aging (>12 months of age) HIV-1 transgenic (Tg) rats. In aging HIV-1 Tg rats, double immunohistochemistry staining revealed abnormal intraneuronal β-amyloid accumulation in the prefrontal cortex (PFC) and hippocampus, relative to F344/N control rats. Notably, in HIV-1 Tg animals, increased β-amyloid accumulation occurred in the absence of any genotypic changes in amyloid precursor protein (APP). Furthermore, no clear amyloid plaque deposition was observed in HIV-1 Tg animals. Critically, β-amyloid was co-localized with neurons in the cortex and hippocampus, supporting a potential mechanism underlying synaptic dysfunction in the HIV-1 Tg rat. Consistent with these neuropathological findings, HIV-1 Tg rats exhibited prominent alterations in the progression of temporal processing relative to control animals; temporal processing relies, at least in part, on the integrity of the PFC and hippocampus. In addition, in post-mortem HIV-1 seropositive individuals with HAND, intraneuronal β-amyloid accumulation was observed in the dorsolateral PFC and hippocampal dentate gyrus. Consistent with observations in the HIV-1 Tg rat, no amyloid plaques were found in these post-mortem HIV-1 seropositive individuals with HAND. Collectively, intraneuronal β-amyloid aggregation observed in the PFC and hippocampus of HIV-1 Tg rats supports a potential factor underlying HIV-1 associated synaptodendritic damage. Further, the HIV-1 Tg rat provides a biological system to model HAND in older HIV-1 seropositive individuals.

## 1. Introduction

The life expectancy of individuals living with human immunodeficiency virus type 1 (HIV-1) dramatically increased following the advent of combination antiretroviral therapy (cART; [1,2]). Indeed, HIV-1 seropositive individuals 50 years of age and older account for approximately 30–50% of all HIV-1 seropositive individuals in high-resource countries [3]; a prevalence which is expected to reach 73% by 2030 [4]. Critically, older HIV-1 seropositive individuals exhibit a higher frequency of neurocognitive deficits relative to their younger counterparts [5,6,7] underscoring the importance of an investigation of the neuropathological mechanisms underlying these disorders.

Synaptodendritic damage [8,9] and spine dysmorphology/loss [10,11,12] have been implicated as key neural mechanisms underlying HAND in HIV-1 seropositive individuals. Fundamentally, synaptic damage, measured using the presynaptic protein synaptophysin and/or the dendritic microtubule activation protein 2, correlates with the severity of neurocognitive impairments [8,13]. Furthermore, multiple biological systems utilized to model HAND exhibit prominent synaptodendritic damage and/or spine dysmorphology/loss (e.g., Tat transgenic (Tg) mice: [14,15]; gp120 Tg mice: [16,17]; HIV-1 Tg rat: [18,19,20,21]; chimeric HIV rat: [22]); alterations that generalize across brain regions (e.g., prefrontal cortex (PFC), nucleus accumbens (NAc), and hippocampus) and ages (e.g., 4 months, 14–17 months, and 20 months of age). However, the factors underlying (e.g., β-amyloid) HIV-1 associated synaptodendritic damage remain elusive [23,24].

Toxic β-amyloid proteins have deleterious effects on neurons, including synaptodendritic loss and spine dysmorphology (e.g., [25,26]) affording a potential mechanism underlying synaptodendritic damage in HIV-1. β-amyloid proteins are formed following the proteolysis of the amyloid precursor protein (APP) along either the nonamyloidogenic or the amyloidogenic pathway (for review, [27]). First, APP is cleaved by either α- (non-amyloidogenic) or β- (amyloidogenic) secretase releasing soluble APPα and soluble APPβ, respectively, from the cell surface. C-terminal fragments of either 83-amino acids (α-secretase; C83) or 99-amino acids (β-secretase; C99) afford substrates for γ-secretase. During amyloidogenic processing, the cleavage of C99 by γ-secretase yields either extracellular β-amyloid peptides of varying lengths (e.g., 51-30 amino acid residues) or the APP intracellular domain. Further cleavage of β-amyloid peptides results in the generation of the main final forms of β-amyloid, including β-amyloid_40_ (Aβ40) and β-amyloid_42_ (Aβ42; [28,29]). Although Aβ40 is the most abundant isoform in the brain [30], Aβ42 is predominant in neuritic plaques (e.g., [31,32]).

Thus, the present study investigated protein aggregates (i.e., β-amyloid) as a potential neuropathological mechanism underlying synaptic dysfunction in HIV-1. First, β-amyloid protein aggregates were assessed in the brains of aging (>12 months of age) HIV-1 transgenic (Tg) and F344/N control rats. The HIV-1 Tg rat expresses seven of the nine HIV-1 genes (deletion of the *pol* and *gag* genes) constitutively throughout development [33] and affords a biological system to model age-related disease progression [20]. Second, the nature of β-amyloid accumulation (i.e., intraneuronal vs. extracellular plaques) was examined in the post-mortem brains of HIV-1 seropositive individuals with HAND. Examination of β-amyloid protein accumulation and its co-localization with neurons affords an opportunity to understand the fundamental factors underlying HIV-1 associated synaptodendritic damage.

## 2. Materials and Methods

### 2.1. Experiment 1: HIV-1 Transgenic Rats

All animals were housed and cared for in AAALAC-accredited facilities according to guidelines established by the National Institutes of Health. The protocols were approved by the Institutional Animal Care and Use Committee (IACUC) at the University of South Carolina (Federal Assurance #D16-00028).

#### 2.1.1. Neuroanatomical Assessments

##### Animals

Aging (>12 months of age) Fischer HIV-1 Tg rats and F344/N control rats were pair-housed in a controlled environment. F344/N control animals were procured from Envigo Laboratories (Indianapolis, IN, USA), whereas HIV-1 Tg animals were bred by housing a control female and HIV-1 Tg male together at the University of South Carolina. Animals were maintained under a 12:12 light/dark cycle with ad libitum access to food (Pro-Lab Rat, Mouse, Hamster Chow #3000) and water.

##### Immunofluorescence Staining

Animals (HIV-1 Tg: male, *n* = 4; female, *n* = 4; F344/N Control: male, *n* = 4; female, *n* = 4) were deeply anesthetized using sevoflurane (Abbot Laboratories, North Chicago, IL, USA) and transcardially perfused with 4% paraformaldehyde. After perfusion, brains were removed, post-fixed overnight in 4% chilled paraformaldehyde, and sectioned using a vibratome (100 µm thick coronal slices). Brain sections were incubated with either the Alexa Fluor^®^ 488 anti-beta Amyloid 1-42 rabbit monoclonal antibody (Cat. No. ab224026, Abcam, Waltham, MA, USA), Alexa Fluor^®^ 594 anti-NeuN rabbit monoclonal antibody (Cat. No. ab207279, Abcam, Waltham, MA, USA), or anti-Amyloid Precursor Protein rabbit monoclonal antibody (Cat. No. ab208744, Abcam, Waltham, MA, USA). Fluorescent images were acquired using a Nikon D-Eclipse C1 inverted fluorescence microscope. Analyses were conducted by evaluating the intensity of immunohistochemistry (IHC) staining using NIS-Elements BR3.10 software (Nikon, Melville, NY, USA), whereby the experimenter was blind to both genotype and sex.

##### Neuronal Labeling

Methodological details for ballistic labeling were previously described in detail [34]. Briefly, Tefzel tubing (IDEX Health Sciences, Oak Harbor, WA, USA) was coated with polyvinylpyrrolidone (PVP). DiOlistic cartridges were prepared using 170 mg tungsten beads (Bio-Rad, Hercules, CA, USA) and lipophilic dye DiI (Invitrogen, Carlsbad, CA, USA), which were dissolved in 99.5% pure methylene chloride (Sigma-Aldrich, St. Louis, MO, USA), and mixed thoroughly. Approximately 100 μL of the bead solution was pipetted onto a standard glass slide and 150 μL DiI was added on top. The air dried bead/dye mixture was suspended in deionized H_2_O, sonicated to homogenize, added to the PVP-coated Tefzel tubing, and dried under a nitrogen flow (0.4 LPM) for 30 min. Finally, the Helios gene gun (Bio-Rad, Hercules, CA, USA) was loaded with the previously prepared PVP-coated Tefzel tubing cartridges. The Dil/tungsten beads within the cartridges were delivered to the tissue sections using the Helios gene gun system. Helium gas pressure was set to 100 psi and brain slices were placed approximately 2.5 cm away from the barrel of the Helios gene gun. DiOlistically labeled tissue sections were mounted onto glass slides using Pro-Long Gold Antifade reagent (Cat. No. D1306, Fisherscience, MA, USA), coverslipped, and stored in the dark at 4 °C. Confocal images were obtained within 48 h of DiOlistic labeling.

#### 2.1.2. Neurocognitive Assessments

##### Animals

Gap-prepulse inhibition (gap-PPI), tapping the cognitive domain of temporal processing, was evaluated in Fischer F344/N (*n* = 20 litters) and HIV-1 Tg (*n* = 17 litters) animals. Animals were procured in litters (F344/N Control, *n* = 20 litters; HIV-1 Tg, *n* = 17 litters) from Harlan Laboratories, Inc. (Indianapolis, IN, USA), arriving at the animal colony between postnatal day (PD) 7 and PD 9. HIV-1 Tg and control animals were sampled from each litter, yielding, HIV-1 Tg: male, *n* = 37, female, *n* = 33 and Control: male *n* = 34; female, *n* = 33. Animals (HIV-1 Tg: *n* = 14; Control: *n* = 10) exhibiting health issues were humanely sacrificed prior to the completion of the study. Animals were placed on food restriction at approximately PD 60, with the goal of maintaining approximately 85% body weight, during the beginning of a concurrently run operant task. Once animals successfully acquired the operant task (PD 100-PD 277), rodent food (Pro-Lab Rat, Mouse, Hamster Chow #3000) was available ad libitum. Water was available ad libitum.

##### Apparatus

The startle platform (SR-Lab Startle Reflex System, San Diego Instruments, Inc., San Diego, CA, USA) was enclosed in an isolation cabinet (external dimensions: 10 cm thick, double-walled, 81 × 81 × 116-cm; Industrial Acoustic Company, Inc., Bronx, NY, USA) that afforded sound attenuation (30 db(A)) relative to the external environment. Within the testing chamber, the ambient sound level was 22 db(A). Thirty cm above the Plexiglas test cylinder was a high-frequency loudspeaker of the SR-Lab system (model#40-1278B, Radio Shack, Fort Worth, TX, USA), which was utilized for the delivery of all auditory stimuli. Deflections of the Plexiglas test cylinder were converted into analog signals based on a piezoelectric accelerometer attached to the bottom of the cylinder. Following the digitation (12 bit A to D, recorded at a rate of 2000 samples/sec) of response signals, they were saved to a hard disk. The SR-LAB Startle Calibration System was utilized to calibrate response sensitivities.

##### Procedure

A longitudinal experimental design was utilized to assess the progression of temporal processing using the gap-prepulse inhibition experimental paradigm. HIV-1 Tg and control animals were tested for gap-PPI of the auditory startle response beginning at PD 240. Assessments were conducted every 60 days through PD 540. The methodology for the assessment of gap-PPI is similar to our prior publication [35]. In brief, the test session, which was approximately 20 min in duration, began with a 5 min acclimation period in the dark with 70 db(A) background white noise. Subsequently, six pulse-only ASR trials were utilized for habituation and separated by a 10 sec intertrial interval (ITI). Thirty-six testing trials were presented in six-trial blocks interdigitated using a Latin Square experimental design with a variable ITI (15–25 s. A 20 msec gap in background white noise preceded the auditory startle stimulus (100 db(A) intensity with a 20 msec duration) at interstimulus intervals (ISIs) of 30, 50, 100, and 200 msec. The gap-PPI assessment included two control trials, including both the 0 and 4000 msec ISI, providing a reference ASR within the assessment. Analyses were conducted on the peak ASR amplitude values.

### 2.2. Experiment 2: Post-Mortem HIV-1 Seropositive Individuals with HAND

Autopsy human brain tissues (*n* = 9) were provided by the National NeuroAIDS Tissue Consortium (NNTC). Study participants were HIV-1 seropositive individuals with symptomatic HAND that had tissue samples from both the dorsolateral prefrontal cortex (dlPFC; Brodmann’s Area 9 [36]; *n* = 9) and hippocampus (dentate gyrus; *n* = 3). Participants died between 55 and 74 years of age. Additional demographic information is available in Supplementary Table S1. The NNTC Data Coordinating Center (DCC) approved the specimen application (Request # R703).

#### 2.2.1. Neuroanatomical Assessments

##### Immunofluorescence Staining

Human brain tissues were sectioned using a cryostat (50 µm thick coronal slices) and incubated overnight at 4 °C with either the Alexa Fluor^®^ 488 Anti-beta Amyloid 1-42 antibody (Cat. No. ab224026, Abcam, Waltham, MA, USA), Alexa Fluor^®^ 594 Anti-NeuN antibody (Cat. No. ab207279, Abcam, Waltham, MA, USA), or PE Anti-Amyloid Precursor Protein antibody (Cat. No. ab208744, Abcam, Waltham, MA, USA). Fluorescent images were acquired using a Nikon D-Eclipse C1 inverted fluorescence microscope. The fluorescence signal was analyzed using NIS-Elements BR3.10 software.

##### Thioflavin-S Staining

Brain sections were immersed in a 1% Thioflavin-S (Cat. No. T1892, MilliporeSigma, Burlington, MA, USA) solution for 2 min at room temperature and differentiated in 70% ethanol until the sections were clear. Sections were washed with deionized water and mounted with Pro-Long Gold Antifade reagent.

### 2.3. Statistical Analysis

Data were analyzed using independent samples *t*-test (SPSS Statistics 27, IBM Corp., Somer, NY, USA), analysis of variance (ANOVA; SPSS Statistics 27), or regression statistical techniques (GraphPad Prism 5.02, GraphPad Software, Inc., La Jolla, CA, USA). Figures were created using GraphPad Prism 5. Statistical significance was established at an alpha level of *p* ≤ 0.05. Partial eta squared (η_p_^2^) is presented as a measure of effect size.

IHC intensity data for β-amyloid or APP were analyzed using an ANOVA, whereby genotype (HIV-1 Tg versus Control) and sex (male versus female) served as between-subjects factors. The measured intensity of β-amyloid in the hippocampal CA3 region was transformed using a square root transformation.

The progression of temporal processing was analyzed using regression statistical techniques. Given the nested experimental design (i.e., rats within litters), individual observations were analyzed using litter means and standard errors, dependent upon biological sex. Additionally, the mean series imputation method was used for all censored data.

## 3. Results

### 3.1. Experiment 1: HIV-1 Transgenic Rats

#### 3.1.1. Neuroanatomical Assessments

**Figure 1:**
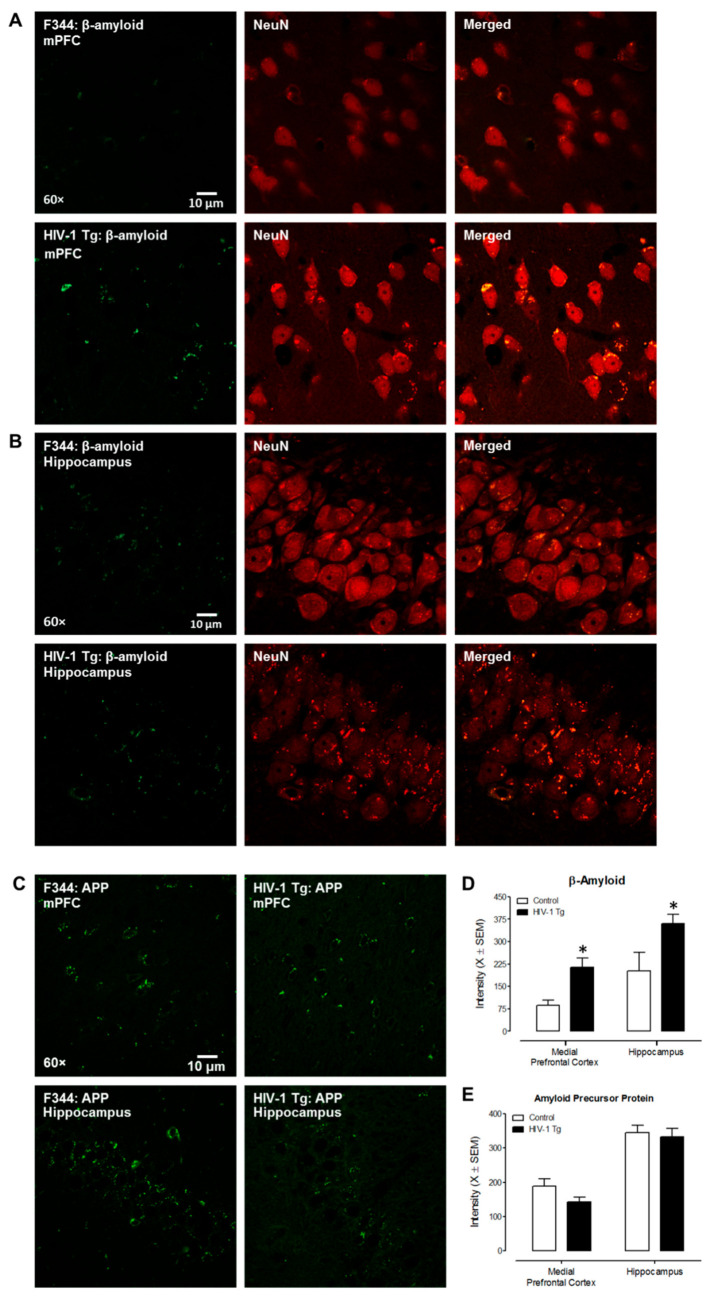
IHC double staining revealed an abnormal intraneuronal accumulation of β-amyloid in the HIV-1 Tg rat. (**A**,**B**) Representative confocal images of β-amyloid expression and co-localization with NeuN (neuronal marker) in the mPFC and CA3 area of hippocampus in F344/N and HIV-1 Tg rats. The Alexa 488 green fluorescence indicates expression of β-amyloid; the Alexa 594 red fluorescence represents NeuN signals. (**C**) Representative images of amyloid precursor protein (APP) expression in the mPFC and hippocampal CA3 region in HIV-1 Tg and F344/N rats. APP expression is indicated by green fluorescence. (**D**) HIV-1 Tg rats exhibited abnormal accumulation of β-amyloid in both the mPFC and hippocampal CA3 region relative to control animals. (**E**) Statistical evaluation of APP in the mPFC and hippocampal CA3 areas compared to control rat. Statistically significant (*p* ≤ 0.05) differences between HIV-1 Tg and control animals are indicated using an *.

IHC (Figure 1) was used to detect the expression of β-amyloid and APP in the medial prefrontal cortex (mPFC) and hippocampal CA3 region of aging HIV-1 Tg and F344/N control rats. With regards to β-amyloid, HIV-1 Tg animals exhibited abnormal accumulation in both the mPFC (main effect of genotype: F(1,15) = 12.6, *p* ≤ 0.004, η_p_^2^ = 0.513) and hippocampal CA3 region (main effect of genotype: F(1,15) = 5.1, *p* ≤ 0.044, η_p_^2^ = 0.296) relative to F344/N control rats (Figure 1D). With regards to APP, no statistically significant genotype and/or sex differences (*p* > 0.05) were observed in either the mPFC or CA3 region of hippocampus (Figure 1E).

**Figure 2:**
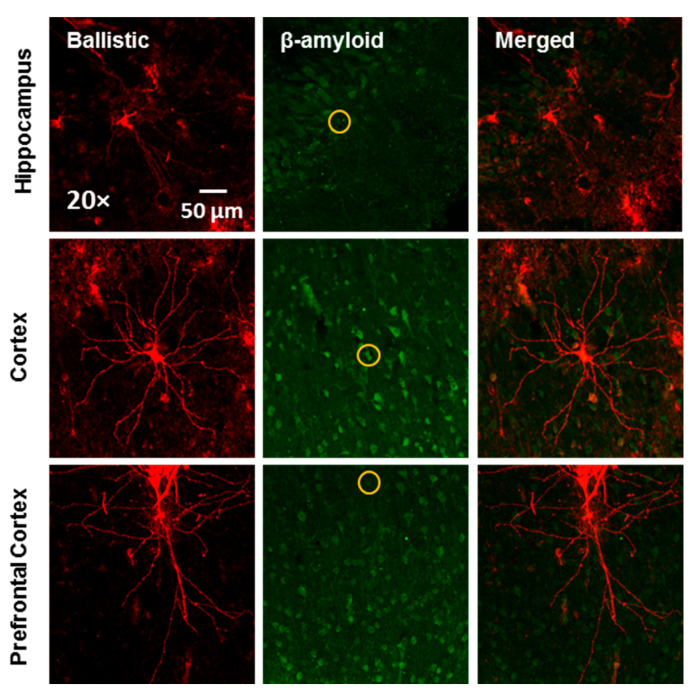
Co-localization of DiOlistically labeled neurons and β-amyloid immunostaining in the hippocampus, cortex, and prefrontal cortex. The yellow circle indicated the DiOlistically labeled neuron co-localizing with β-amyloid immunostaining positive signal.

Furthermore, two methods (i.e., IHC double staining and DiOlistic labeling) were utilized to evaluate the location of β-amyloid accumulated. First, double staining of β-amyloid and NeuN, a neuronal marker, supports a strong co-localization of β-amyloid signals and neurons in both the mPFC and hippocampal region (Figure 1A,B). Second, DiOlistic labeling was also performed using ballistic techniques to confirm the co-localization between β-amyloid and hippocampal and/or cortical neurons (Figure 2); observations which further support β-amyloid accumulation as a potential mechanism underlying synaptic alterations in the HIV-1 Tg rat.

#### 3.1.2. Neurocognitive Assessments

**Figure 3:**
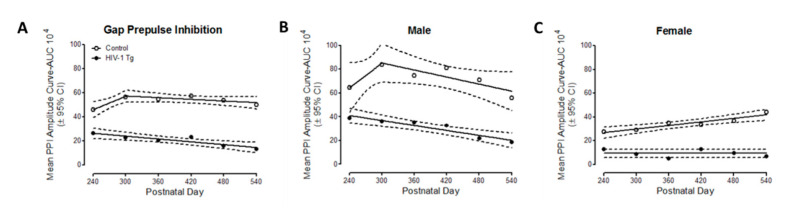
Neurocognitive assessments of temporal processing were evaluated using gap-prepulse inhibition (gap-PPI). The mean peak ASR amplitude response curve for gap-PPI was used to calculate prepulse inhibition in HIV-1 Tg and control rats from PD 240 to PD 540. (**A**) At the genotypic level, HIV-1 Tg animals exhibited a profound alteration in the progression of temporal processing relative to control animals. (**B**,**C**) Fundamentally, both male (**B**) and female (**C**) HIV-1 Tg animals displayed prominent alterations in the progression of temporal processing; the magnitude of these alterations was influenced by biological sex.

In gap-PPI, the area of the inflection of the ASR response curve (a measure of prepulse inhibition), was utilized to examine the progression of temporal processing in HIV-1 Tg and control rats from PD 240 to PD 540 (Figure 3). HIV-1 Tg animals, relative to controls, displayed a prominent alteration in the progression of temporal processing (Figure 3A). For control animals, a segmental linear regression provided a well-described fit, with a linear increase in maximal prepulse inhibition observed through approximately PD 300, followed by a subsequent decline (*R*^2^ = 0.85). In sharp contrast, a first-order polynomial with a negative slope (i.e., β_1_ = −392.9 ± 239.6 (X ± 95% confidence interval)) provided a well-described fit for HIV-1 Tg rats (*R*^2^ = 0.83). The magnitude of alterations in the progression of temporal processing, however, was significantly influenced by the factor of biological sex (Figure 3B,C).

Complementary analyses of each genotype were conducted to determine the locus of these interactions. In male rats (Figure 3B), a segmental linear regression provided a well-described fit for control animals (*R*^2^ = 0.76) with maximal inhibition observed at PD 300, followed by a subsequent decline. Temporal processing in male HIV-1 Tg rats, however, was well-described by a first-order polynomial with a negative slope *R*^2^ = 0.96; β_1_ = −392.9 ± 344.12 (X ± 95% confidence interval)). In female rats (Figure 3C), a first-order polynomial provided an appropriate fit for the development of temporal processing in control rats (*R*^2^ = 0.87). However, female HIV-1 Tg rats failed to exhibit any significant development in temporal processing from PD 240 to PD 540, evidenced by a horizontal fit. Aging HIV-1 Tg rats, therefore, displayed prominent alterations in the progression of temporal processing, with more significant deficits observed in female rats.

### 3.2. Experiment 2: Post-Mortem HIV-1 Seropositive Individuals with HAND

**Figure 4:**
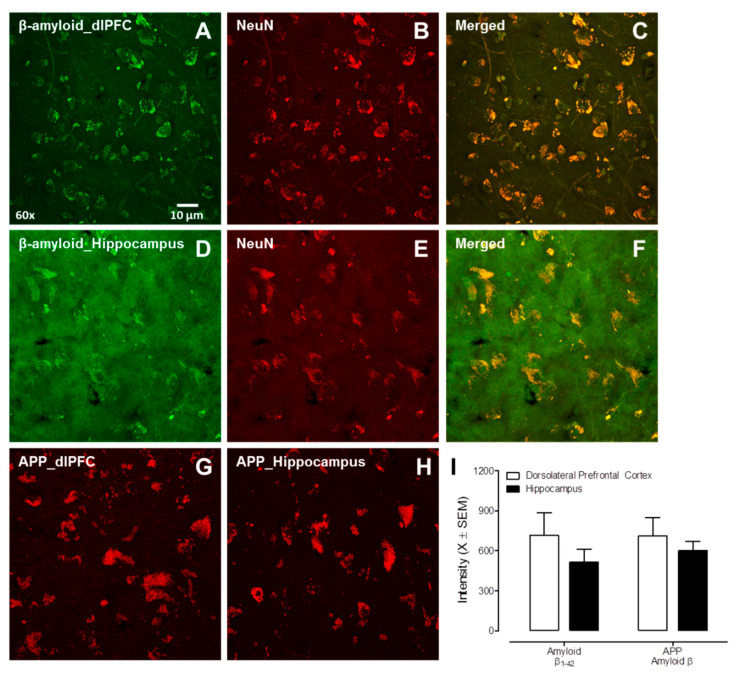
β-amyloid accumulation in the dorsolateral PFC (dlPFC) and hippocampal dentate gyrus from human autopsy HIV-infected cases with HAND. (**A**–**F**) Representative images of double staining of β-amyloid with NeuN (a neuronal marker) in the dlPFC (**A**–**C**), and in the hippocampus from human autopsy (**D**–**F**). (**G**,**H**) Confocal images of amyloid precursor protein expression in the dlPFC and hippocampus. (**I**) Quantification of β-amyloid and amyloid precursor protein expression in the dlPFC and hippocampus.

The nature of β-amyloid accumulation (i.e., intraneuronal vs. extracellular plaques) was examined in the dorsolateral PFC (Area 9, *n* = 9) and hippocampal dentate gyrus (*n* = 3) of post-mortem HIV-1 seropositive individuals with HAND. Both β-amyloid 1-42 and APP were observed in the dlPFC and hippocampus of HIV-1 seropositive individuals (Figure 4). Critically, there was no significant β-amyloid plaque deposition in either the dlPFC or hippocampus, evidenced by the absence of thioflavin-s staining. Meanwhile, the double staining of β-amyloid with NeuN (a neuronal marker) suggested that β-amyloid accumulation occurred intraneuronally. Collectively, aged HIV-infected individuals with HAND exhibited intraneuronal β-amyloid accumulation in the absence of any significant β-amyloid plaques.

## 4. Discussion

Intraneuronal β-amyloid accumulation was observed in the frontal cortex and hippocampus in both HIV-1 Tg rats and HIV-1 seropositive individuals with HAND. Notably, in HIV-1 Tg rats, increased β-amyloid accumulation occurred in the absence of any genotypic changes in APP. Consistent with these neuropathological findings, HIV-1 Tg rats exhibited prominent alterations in cognitive processes (i.e., temporal processing) dependent upon hippocampal and PFC function. Critically, the intraneuronal nature of β-amyloid accumulation in HIV-1 seropositive individuals is consistent with previous reports (e.g., [37,38]) and resembled observations in the HIV-1 Tg rat. Collectively, intraneuronal β-amyloid accumulation observed in the frontal cortex and hippocampus of both HIV-1 seropositive individuals and the HIV-1 Tg rat supports a potential factor underlying the HIV-1 associated synaptodendritic alterations.

HIV-1 viral proteins may underlie the abnormal intraneuronal accumulation of β-amyloid. Specifically, HIV-1 viral proteins may alter β-amyloid synthesis and/or β-amyloid degradation [23,38,39,40]; alterations that would decrease the clearance of β-amyloid. First, the HIV-1 transactivator of transcription (tat) and envelope glycoprotein gp120 (gp120) may promote the synthesis, secretion, and accumulation of β-amyloid [23,39]. Second, tat and/or tat-derived peptides may inhibit neprilysin [39,41,42], a key enzyme for β-amyloid degradation [43]. In addition, it is well-recognized that the blood–brain barrier (BBB) is compromised by HIV-1 viral proteins (for review, [44]); dysfunction which may influence β-amyloid homeostasis. For example, HIV-1 particles increased the release of BBB-derived extracellular vesicles and increased the β-amyloid cargo load in extracellular vesicles [45].

In addition to these neuropathological findings, HIV-1 Tg rats exhibited prominent alterations in the progression of temporal processing relative to control animals. In preclinical biological systems, temporal processing is often evaluated using prepulse inhibition (PPI) of the auditory startle response (ASR; [46,47]); the gap-PPI experimental paradigm utilized in the present study is based on the modification of PPI [48]. Specifically, both PPI and gap-PPI rely upon the presentation of a discrete prestimulus and a startling stimulus [49]. However, whereas the discrete prestimulus is added (e.g., tone) in PPI, the discrete prestimulus is removed (e.g., a gap in background noise) in gap-PPI. Indeed, in gap-PPI, the presentation of a discrete prestimulus 30 to 200 msec prior to the startling stimulus elicits a pronounced reduction in startle response [48]. High translational relevance (e.g., via utilization of the eyeblink startle experimental paradigm as in [50]) and the well-established neural circuitry (for review, [51]) illustrate two of the key advantages of utilizing the PPI and gap-PPI experimental paradigms.

Both the PFC and hippocampus [51], brain regions that exhibit abnormal intraneuronal β-amyloid accumulation in aging HIV-1 Tg rats, are fundamentally involved in the regulation of PPI. The serial neural circuit mediating PPI begins by relaying auditory input to the inferior colliculus, which subsequently innervates the superior colliculus. Sensory input from the superior colliculus is then sent to the pedunculopine tegmental nucleus (PPTg). Cholinergic projections from the PPTg to the pontine reticular nucleus are relayed to motor neurons resulting in the elicitation of a startle response. Both the ventral hippocampus [52] and PFC [53,54] send afferents to the nucleus accumbens (NAc), which subsequently innervates the PPTg. Disruption of neurotransmission in either the ventral hippocampus (e.g., [55,56]) or PFC (e.g., [57,58]) lead to prominent reductions in PPI; reductions which resemble those observed in the HIV-1 Tg rat. Given the fundamental role of β-amyloid in neurotransmission (for review, [59]), it is conceivable that the abnormal intraneuronal β-amyloid accumulation may underlie the prominent alterations in the progression of temporal processing observed in HIV-1 Tg animals.

Due to the increasing prevalence of older HIV-1 seropositive individuals, differentiating HAND from other neurodegenerative diseases, including Alzheimer’s disease (AD), is a fundamental concern; the results of the present paper highlight two facets of HAND that differentiate it from AD. First, HIV-1 Tg rats and HIV-1 seropositive individuals with HAND exhibited intraneuronal β-amyloid accumulation in the absence of any significant extracellular β-amyloid plaques. In sharp contrast, one of the salient pathological features of AD is visible, neuritic extracellular plaques [60,61] comprised primarily of β-amyloid [62]. It is noteworthy that diffuse (rather than neuritic) β-amyloid plaques were previously observed in HIV-1 seropositive individuals not receiving cART treatment [63]. In the cART era, when extracellular plaques have been observed in HIV-1 seropositive, they are primarily located in the perivascular regions [37,38]; again, in sharp contrast to the location (i.e., initially in the entorhinal cortex and hippocampus; [64]) of extracellular neuritic plaques associated with AD. Second, temporal processing has been proposed as a key neurobehavioral mechanism underlying neurocognitive impairments associated with HIV-1 [65]. Cross-sectional studies have identified deficits in temporal processing and/or PPI in HIV-1 seropositive individuals with HAND [50] and multiple biological systems utilized to model HAND (e.g., HIV-1 Tg Rat [66,67]; Stereotaxic Injections of Tat [68,69] or gp120 [70]; gp120 Transgenic Mice: [71,72]; Tat Transgenic Mice: [73]). A longitudinal study in the HIV-1 Tg rat revealed alterations in the progression of temporal processing [35,74]; the present study extends these observations, revealing their generalizability via the evaluation of temporal processing using a different experimental paradigm. With regards to AD, however, evidence for alterations in temporal processing has been inconclusive [75]. More broadly, HIV-1 and AD are differentiated by unique neurocognitive profiles, whereby HAND primarily exhibits a “subcortical” pattern (e.g., attention, executive function; for review [76]). Indeed, evaluating six cognitive measures accurately discriminates between milder forms of HAND and AD with high accuracy (i.e., 86%; [77]). There remains, however, a critical need to further delineate similarities and differences in the phenotype of HAND and AD necessitating a biological system to model HAND in older HIV-1 seropositive individuals.

Observations across a multitude of studies, including the present one, support the utility of the HIV-1 Tg rat to model HAND in older HIV-1 seropositive individuals. The HIV-1 Tg rat, originally reported by Reid et al. [33], expresses seven of the nine HIV-1 genes constitutively throughout development; the deletion of *gag* and *pol* renders the HIV-1 Tg rat non-infectious. The contemporary phenotype of the HIV-1 Tg rat, on the F344/N background strain, is healthy through advanced age, with approximately 50% of HIV-1 Tg rats surviving through 21 months of age [78]. Furthermore, HIV-1 Tg rats exhibit intact sensory (i.e., auditory, visual) and gross-motoric system function through advancing age [74] affording an opportunity to evaluate neurocognitive impairments. Indeed, cross-sectional studies have demonstrated that neurocognitive impairments observed in the HIV-1 Tg rat (e.g., attention: [79,80]; executive function: [20,79]; memory: [81,82,83]; preattentive processes/temporal processing: [66,67]) resemble those commonly altered in HIV-1 seropositive individuals on cART [84,85]. Longitudinal studies have further illustrated progressive neurocognitive impairments in the HIV-1 Tg rat through the functional lifespan [20,74]. Prominent sex differences in neurocognitive impairments have been observed in HIV-1 seropositive individuals, whereby female, relative to male, HIV-1 seropositive individuals exhibit greater neurocognitive impairments [86]; findings which have been recapitulated in the HIV-1 Tg rat [20,80]. With regards to potential neural mechanisms underlying HAND, the HIV-1 Tg rat exhibits prominent synaptodendritic damage in multiple brain regions (PFC: [20,21]; Nucleus Accumbens: [18,19,87]); damage which progresses through six months of age [88]. Additionally, as illustrated in the present study, the intraneuronal nature of β-amyloid accumulation in the HIV-1 Tg rat resembled observations in HIV-1 seropositive individuals with HAND. Collectively, the neuropathological and neurocognitive findings of the present study afford additional credence to the utility of the HIV-1 Tg rat as a biological system to model HAND in older HIV-1 seropositive individuals.

Despite the strengths of the present study, a few limitations must be acknowledged. First, the absence of matched human control autopsy brain tissue prevents the determination of whether intraneuronal β-amyloid accumulation in HIV-1 seropositive individuals is abnormal. Given the request for HIV-1 seropositive human autopsy brain tissue from individuals with neurocognitive impairments, we posit that intraneuronal β-amyloid accumulation, in the absence of prominent β-amyloid plaques, reflects brain pathology in the current population. Second, neuroanatomical and neurocognitive assessments were conducted in two separate cohorts of animals; synaptodendritic alterations were not evaluated in the present study. Future studies directly evaluating the relationship between β-amyloid accumulation, synaptodendritic alterations, and neurocognitive function are critical to further enhancing our understanding of HAND pathology.

In conclusion, potential abnormal intraneuronal β-amyloid accumulation supports a potential factor underlying the neural pathogenesis of HAND in the post-cART era. Aging HIV-1 Tg rats exhibited abnormal intraneuronal β-amyloid accumulation in both the PFC and hippocampus. Furthermore, the HIV-1 Tg rat exhibited prominent alterations in temporal processing; a cognitive process that is dependent upon intact PFC and hippocampal. Elucidating a potential factor underlying synaptodendritic alterations in HIV-1 affords a key target for future studies evaluating novel therapeutics.

## Data Availability

All data are available in the main text.

## Supplementary Materials

The following supporting information can be downloaded at: https://www.mdpi.com/article/10.3390/v14061268/s1, Table S1: Information of Manhattan HIV Brain Bank participants.
