# Supplementary material for: Intraneuronal β-Amyloid Accumulation: Aging HIV-1 Human and HIV-1 Transgenic Rat Brain
Source: Viruses. 2022 Jun 10;14(6):1268. doi: 10.3390/v14061268 (PMC9230035; doi:10.3390/v14061268)
Supplement: Supplementary file 1 [file viruses-14-01268-s001.zip › viruses-1702260-supplementary.pdf]

Table S1. Manhattan HIV Brain Bank participants:

PID	age	sex	race	HIV risk	Last VL	Last CD4
10288	67	f	b	sex	<20	598
10214	55	f	h	ivdu	und	141
10321	74	m	w	sex	202	429
10272	61	m	b	sex	<20	374
10229	59	f	b	sex	<20	747
10163	68	m	b/ind	ivdu	und	527
